# Theoretical and experimental study on the detection limit of the micro-ring resonator based ultrasound point detectors

**DOI:** 10.1016/j.pacs.2023.100574

**Published:** 2023-11-29

**Authors:** Youngseop Lee, Qiangzhou Rong, Ki-Hee Song, David A. Czaplewski, Hao F. Zhang, Junjie Yao, Cheng Sun

**Affiliations:** Department of Biomedical Engineering, Northwestern University, Evanston, IL 60208, USA; Department of Mechanical Engineering, Northwestern University, Evanston, IL 60208, USA; Department of Biomedical Engineering, Duke University, Durham, NC 27708, USA; Department of Biomedical Engineering, Northwestern University, Evanston, IL 60208, USA; Center for Nanoscale Materials, Argonne National Laboratory, Argonne, IL 60439, USA; Department of Biomedical Engineering, Northwestern University, Evanston, IL 60208, USA; Department of Biomedical Engineering, Duke University, Durham, NC 27708, USA; Department of Mechanical Engineering, Northwestern University, Evanston IL 60208, USA

**Keywords:** Polymer micro-ring resonator, Ultrasound detector, Photoacoustic imaging, Photoacoustic computed tomography

## Abstract

Combining the diffusive laser excitation and the photoacoustic signals detection, photoacoustic computed tomography (PACT) is uniquely suited for deep tissue imaging. A diffraction-limited ultrasound point detector is highly desirable for maximizing the spatial resolution and the field-of-view of the reconstructed volumetric images. Among all the available ultrasound detectors, micro-ring resonator (MRR) based ultrasound detectors offer the lowest area-normalized limit of detection (nLOD) in a miniature form-factor, making it an ideal candidate as an ultrasound point detector. However, despite their wide adoption for photoacoustic imaging, the underlying signal transduction process has not been systematically studied yet. Here we report a comprehensive theoretical model capturing the transduction of incident acoustic signals into digital data, and the associated noise propagation process, using experimentally calibrated key process parameters. The theoretical model quantifies the signal-to-noise ratio (SNR) and the nLOD under the influence of the key process variables, including the quality factor (Q-factor) of the MRR and the driving wavelength. While asserting the need for higher Q-factors, the theoretical model further quantifies the optimal driving wavelength for optimizing the nLOD. Given the MRR with a Q-factor of 1 × 10^5^, the theoretical model predicts an optimal SNR of 30.1 dB and a corresponding nLOD of 3.75 × 10^−2^ mPa mm^2^/Hz^1/2^, which are in good agreement with the experimental measurements of 31.0 dB and 3.39 × 10^−2^ mPa mm^2^/Hz^1/2^, respectively. The reported theoretical model can be used in guiding the optimization of MRR-based ultrasonic detectors and PA experimental conditions, in attaining higher imaging resolution and contrast. The optimized operating condition has been further validated by performing PACT imaging of a human hair phantom.

## 1 Introduction

Photoacoustic (PA) imaging enables noninvasive volumetric imaging of biological tissues by capturing the endogenous optical absorption contrast [1], [2], [3]. PA employs incident short-pulsed light irradiation to excite molecules through optical absorption. The resulting energy transfer into heat promotes a local tissue vibration, which subsequently generates readily detectable ultrasonic waves. Since the amplitude of the generated ultrasonic waves is determined by the product of the optical absorption coefficient and the optical fluence, a PA image reflects the volumetric optical absorption distribution in the tissue. Because the tissue scattering of ultrasonic waves is two orders of magnitude lower than that of optical scattering, PA is better suited for deep tissue imaging [4], [5]. For the interest of high resolution for deep tissue imaging, optical resolution photoacoustic microscope (OR-PAM) employs a focused incident laser beam to spatially confine the PA generation. The significantly reduced scattering of the ultrasonic waves makes it possible to image deeper into the tissue compared with confocal microscopy at a given optical irradiation wavelength. However, the attainable imaging depth is still constrained by the optical scattering of the incident focused illumination. Photoacoustic computed tomography (PACT) mitigates this issue by exploiting the diffusive light to illuminate deep into the tissues, delivering ultrasonically defined spatial resolution at depths far beyond the optical diffusion regime around 1 mm, which exceeds the reach of conventional ballistic optical imaging modalities [6].

In PACT imaging, the size of the ultrasound detector determines the point spread function when sampling the complex acoustic wavefront, which ultimately determines the lateral resolution [7], [8], [9]. Furthermore, its maximum angular detection range is inversely proportional to the detector size, due to the diffraction nature of the acoustic wave. The angular detection range, the total array aperture size, and the working distance will collectively determine the field-of-view (FOV). Thus, the desire to resolve fine details in PACT images necessitates an ultrasonic point detector with high sensitivity over a broad ultrasound frequency range. Despite their popularity, ultrasound detectors using piezoelectric materials are facing difficulties in scaling down the sensing area to a size comparable with the acoustic wavelength in water. This is largely due to the limited sensitivity per unit area. Furthermore, the optically opaqueness nature of commonly used piezoelectric materials often obstructs the incident PA excitation. Recently, transparent piezoelectric ultrasound detectors [10], [11], [12], [13] have been demonstrated to overcome the limitation of conventional ultrasound detectors in OR-PAM by simplifying coaxial alignment of optic and acoustic paths and realizing integration with other imaging modalities. However, the transparent piezoelectric transducers need further improvements to increase the optical transmission, the detection sensitivity, and frequency bandwidth [14]. The recent emergence of optical-based detectors has thus offered an attractive solution towards miniaturizing the sensing area without compromising the ultrasound detection sensitivity [15], [16]. A variety of optical-based ultrasound detectors being developed have shown promise in greatly improving the detection sensitivity over a wide frequency range. Examples of optical-based ultrasound detectors include free space optics-based sensors [17], [18], [19], [20], prism-based sensors [21], [22], [23], and optical fiber-based sensors [24], [25], [26], [27], [28]. Further exploiting strong optical resonance, integrated photonic devices, including silicon-on-insulator resonators with Bragg gratings [29], [30] and polymer micro-ring resonators (MRRs) [31], [32], [33], [34], significantly reduced the detection limit and sensing areas comparable to or even smaller than the subjecting acoustic wavelength. Among them, a polymer MRR has been proven to be the most versatile choice due to its high sensitivity and frequency bandwidth over a miniaturized form-factor, optical transparency, and low-cost fabrication. It provides a high sensitivity with high Q-factor of ∼10^5^, a broad detection frequency range up to 250 MHz, a small detection area with a size less than 80 µm, and the highly desirable optical transparency. Collectively, the resulting detector has an nLOD of 1.8 × 10^−3^ mPa·mm^2^/Hz^1/2^, which represents more than one order of magnitude improvement compared with all other ultrasonic detectors, as reported in a recent review article [16]. Furthermore, its optical transparency minimizes the interference with the incident photoacoustic excitation beam, allowing photoacoustic detection at increased detection sensitivity and frequency bandwidth. Finally, a scalable nanofabrication method of the polymer MRR based on soft nanoimprinting lithography significantly reduces the fabrication cost and improves the fabrication yield. Over the past decade, its broad detection bandwidth has thus enabled isometric multimodal PAM [35]; its optical transparency and miniature form-factor has granted the development of PA endoscope [36] and smart cranial window for longitudinal in vivo PAM imaging [37]. More recently, its point-like form-factor and high sensitivity allowed the development of deep-tissue high-frequency three-dimensional (3D) PACT with a large field-of-view [38].

Despite its broad applications, the lack of a systematic study of the underlying signal transduction process compromises the ability to fully optimize the operating conditions of MRRs to maximize their ultrasound detection sensitivity. To address this issue, we develop a comprehensive signal-transduction model that accounts for all the major noise factors influencing the detection limit of the MRR-based ultrasound detector, with the key parameters being measured experimentally. The nLOD is theoretically and experimentally investigated under the varying Q-factors and the driving wavelengths of MRR-based ultrasound detector. The optimized operating condition has been further validated in the context of PACT imaging using a phantom consisting of a human hair sample.

## 2 Material and methods

### 2.1 PA signal transduction in the MRR-based PACT system

**Fig. 1:**
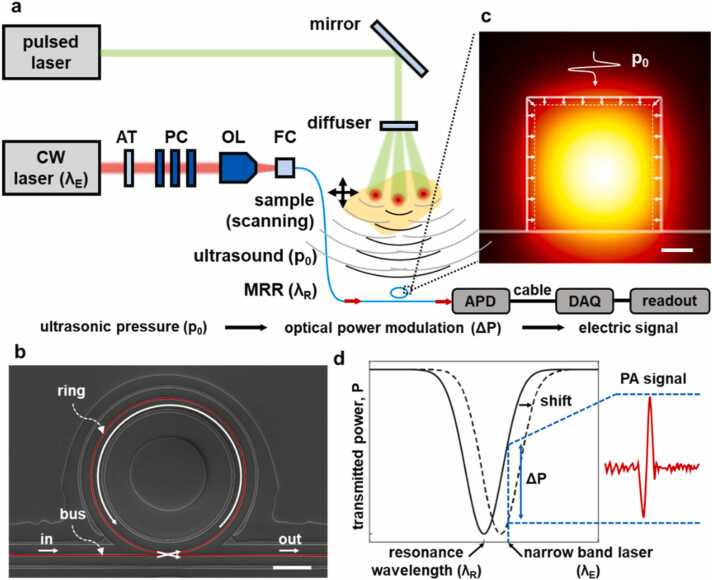
a) A schematic illustration of micro-ring resonator (MRR) based photoacoustic computed tomography (PACT) signal acquisition. AT: attenuator, PC: polarization controller, OL: objective lens, APD: avalanche photodetector, FC: fiber connector, DAQ: data acquisition. b) SEM image of the MRR with a radius of 40 µm. scale bar: 20 µm. c) A numerically calculated electric field in the ring waveguide for the fundamental transverse electric (TE) mode. Ultrasonic pressure (p_0_) changes the resonance spectrum of the ring waveguide by changing the dimension of the waveguide and the refractive index of the materials. scale bar: 200 nm. d) A principle of PA signal transduction through the MRR. A shift of the MRR resonance spectrum by ultrasonic pressure induces the modulation of transmitted optical power through the MRR for the fixed driving wavelength (λ_E_). The optical signal can be transduced to an electrical signal through the PD.

A representative MRR-based PACT system used in this study is schematically illustrated in Fig. 1a. A ns-pulsed Nd: YAG laser (Q-smart 850, *Quantel*) with a wavelength of 532 nm and a pulsed repetition frequency of 10 Hz is used as the PA excitation source. The laser beam was expanded and homogenized using an optical diffuser (DG10–220-MD, *Thorlabs*) and then illuminated onto the sample. A second tunable narrow band laser (TLB-6172, *Newport*) is used to drive the MRR near its resonance for detecting the generated PA pressure waves. The polystyrene MRR with a radius of 40 µm was fabricated on a quartz substrate using a soft-nanoimprinting lithography process (Fig. 1b) [37], [38]. The MRR was butt-coupled to a single mode fiber (SMF, S630-HP, *Thorlabs*) as an input port and a multimode fiber (MMF, GIF625, *Thorlabs*) as an output port. The narrow band laser output was conditioned using an attenuator (AT) and a polarization controller (PC) and then coupled into the input port of a MRR using an objective lens (OL). The detection of the PA signal constitutes multiple sequential signal transduction steps. First, an incident pulsed laser photoacoustically generates the ultrasonic pressure wave, which propagates towards the MRR-based ultrasound detector. The incident pressure wave deforms the polystyrene MRR waveguide and results in a change of its effective optical pathlength due to the elasto-optic effect, and subsequently causes a wavelength shift in MRR’s resonance modes (Fig. 1c). The use of a narrow band laser allows the shift in the resonance wavelength to be monitored as the intensity modulation of the transmissive optical signal at the output port (Fig. 1d), which is then converted into a voltage signal using a photodiode detector (PD, APD430A, *Thorlabs*) and then digitized using a data acquisition (DAQ) card (ATS9350, *Alazar*) at a sampling rate of 500 MHz.

### 2.2 Comprehensive theoretical model

**Fig. 2:**
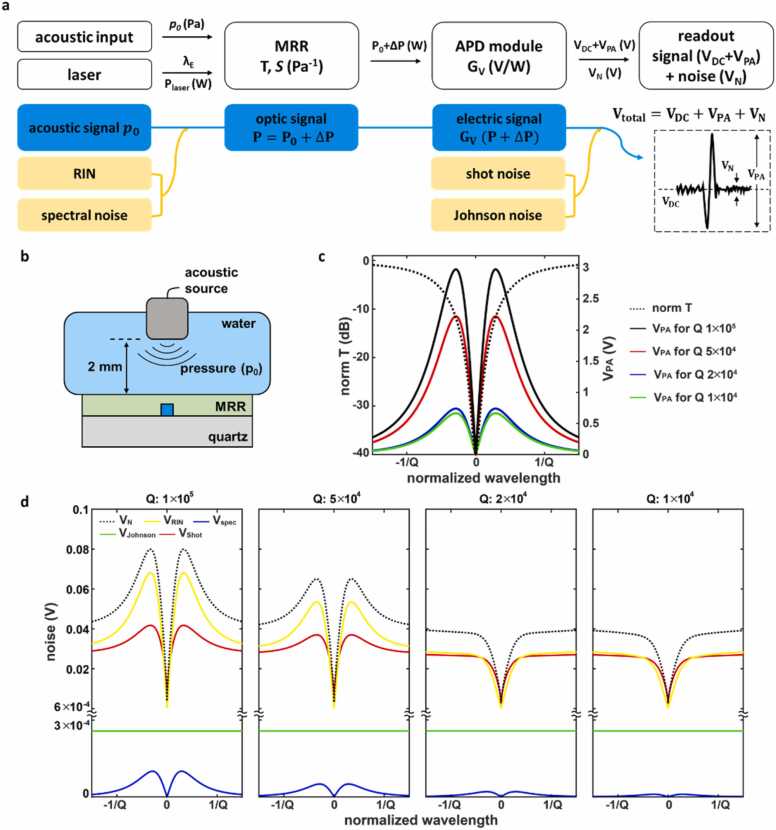
a) A schematic flow of MRR based PA signal transduction and major noises in the system. *p*_*0*_: input acoustic pressure. λ_E_: driving wavelength. P_0_: Initial optical power through the MRR at λ_E_. ΔP: Optical power variation for *p*_*0*_. G_V_: photoelectric sensitivity of the PD module. V_DC_: DC electric signal. V_PA_: PA signal. V_N_: total noise. V_DC_, V_PA_, and V_N_ correspond to DC bias voltage, peak-to-peak voltage, and root-mean-square voltage in the experimentally measured readout. b) A schematic illustration of the measurement setup of the MRR detection sensitivity. c) The normalized transmission spectrum of the MRR is generated based on a Lorentzian curve (dash line). The calculated absolute value of the acoustic signal (solid line) for the MRR with a Q-factor of 1 × 10^5^, 5 × 10^4^, 2 × 10^4^, and 1 × 10^4^ and an input pressure of 1142 Pa. d) Calculated noise values for a Q-factor of 1 × 10^5^, 5 × 10^4^, 2 × 10^4^, and 1 × 10^4^ and an input pressure of 1142 Pa.

The above-mentioned signal transduction processes are summarized into a flow-chart shown in Fig. 2a. The signals and the noises associated with each of the transduction steps are respectively represented in blue and yellow colors. As illustrated in Fig. 1d, coupling the narrow-band laser at the driving wavelength λ_E_ and the power P_laser_ into the MRR with a resonance wavelength at λ_R_ generates a base-line transmissive optical power P = P_0_. In the event of PA detection, the acoustic pressure *p*_*0*_ acting on the MRR shifts the resonance from λ_R_ to λ_R_', which changes the optical transmission at λ_E_ to P_0_ + ΔP, where ΔP represents the changes in the optical transmission. The baseline optical transmission P_0_ is the product of the transmittance of the MRR at λ_E_ and the incident laser power P_laser_. The optical signal variation, ΔP, is proportional to the input pressure and the detection sensitivity of the MRR. Therefore, the transmissive optical signal is defined as

(1) $$P=P_{0}+∆P=T(\lambda_{E})P_{\mathrm{laser}}+p_{0}SP_{\mathrm{laser}},$$

where T(λ_E_) is the transmittance of the MRR at the driving wavelength λ_E_, *S* is the calibrated transmittance sensitivity of the detecting pressure change through the MRR. The calibrated detection sensitivity *S* is defined by

(2) $$S=C\frac{\mathrm{dT}(\lambda )}{dp}=C\frac{dn_{\mathrm{eff}}}{dp}\frac{d\lambda }{dn_{\mathrm{eff}}}\frac{\mathrm{dT}(\lambda )}{d\lambda },$$

where *p* is the pressure, n_eff_ is the effective refractive index of the guided mode, and the coefficient *C* will be determined experimentally [33], [39]. The first term $dn_{\mathrm{eff}}/dp$ defines the pressure-induced change of the effective refractive index in the polymeric waveguide, which is collectively determined by the cross-sectional area of the waveguide, as well as the Young’s modulus and elasto-optic coefficient of the waveguide materials. Using the commercial finite element analysis software (COMSOL Multiphysics Version 6.0, *COMSOL Inc.*), we determined the $dn_{\mathrm{eff}}/dp$ to be − 5 × 10^−5^ MPa^−1^ in the current MRR configuration. The second term $d\lambda /dn_{\mathrm{eff}}$ can be approximated as $\lambda /n_{\mathrm{eff}}$ = 780 nm/1.5 = 520 nm under a small perturbation in $n_{\mathrm{eff}}$. The third term dT(λ)/dλ can be defined as the slope of the resonance spectrum at λ_E_, Note that the resonance spectrum of an MRR can be approximated using a Lorentzian form with a Q-factor defined by $\lambda_{R}/\mathrm{FWHM}$, where FWHM is the full width at half maximum of the resonance curve [40], and the minimum transmittance of 0.01 at $\lambda_{R}$.

Then, the output optical signal from the MRR is converted into a photocurrent:

(3) $$i_{\det}=G_{A}P=G_{A}T(\lambda_{E})P_{\mathrm{laser}}+G_{A}p_{0}SP_{\mathrm{laser}},$$

where G_A_ is the responsivity of the PD. The photocurrent *i*_det_ is then converted into a voltage with the transimpedance amplifier as an integral part of the PD. The PD used in this study has a responsivity G_A_ of 53 AW^−1^ and a transimpedance gain R_F_ of 10 kVA^−1^ [41].

(4) $$\begin{array}{c} V_{\mathrm{out}}={(R}_{F}+R_{L})i_{\det} \end{array},$$

where $R_{F}$ is the transimpedance gain and $R_{L}$ is the load resistance. As the transimpedance gain $R_{F}$ is in general much greater than the load resistance R_L_, the voltage output from the PD can be approximated as

(5) $$V_{\mathrm{out}}\approx i_{\det}R_{F}=G_{A}R_{F}T\left(\lambda_{E}\right)P_{\mathrm{laser}}+G_{A}R_{F}p_{0}SP_{\mathrm{laser}}=V_{\mathrm{DC}}+V_{\mathrm{PA}}$$

The first term in Eq. (5) represents the DC components originated from the based-line optical transmission of the MRR and thus noted as V_DC_, while the second term represents the oscillating components proportional to the time-varying PA-induced pressure wave and thus noted as V_PA_. The digitization of the voltage signal creates the final PA induced acoustic waveform in the data form.

While the incident acoustic pressure wave has been converted and amplified into the final data form, a wide variety of noises are also propagated and amplified through the same signal transduction process (Fig. 2a). The limit of detection (LOD) of an MRR is ultimately determined by the SNR of the final recorded signals. Thus, we have identified and included the major noise factors in the forms of optical noises and electrical noises in the theoretical model. Firstly, the optical noises originate from the power fluctuation and spectral instability of the narrow-band laser used to drive the MRR and the MRR resonance drift due to ambient temperature fluctuations. Secondly, the electrical noises are induced when the PD transduces the optical signals into the voltage signals, including shot noise and Johnson noise.

Contributions of major noise factors in the final voltage signals are theoretically calculated and quantified as follows. Power fluctuation of the narrow-band laser is generally defined as the relative intensity noise (RIN) = $\sqrt{S_{\mathrm{RIN}}(f)∆f}$, where $S_{\mathrm{RIN}}(f)$ is the power spectral density and ∆f is the system frequency bandwidth [42]. As the laser power fluctuation propagates through the signal transduction process described in Eq. (5), the resulting variation in the final voltage output is

(6) $$V_{\mathrm{RIN}}=G_{A}R_{F}T(\lambda_{E})\Delta P_{\mathrm{laser}}+G_{A}R_{F}p_{0}S\Delta P_{\mathrm{laser}}=\mathrm{RIN}(G_{A}R_{F}T(\lambda_{E})P_{\mathrm{laser}}+G_{A}R_{F}p_{0}SP_{\mathrm{laser}})$$

On the other hand, the statistic spectral instability (Δλ_spec_) of the narrow-band laser with a linewidth of $\Delta f_{B}$ is defined as $\Delta f_{B}\lambda^{2}/c$. As the narrow-band laser is tuned into resonance with the MRR as illustrated in Fig. 1c, the resulting variation is

(7) $$V_{\mathrm{spec}}=G_{A}R_{F}P_{\mathrm{laser}}\frac{\mathrm{dT}(\lambda )}{d\lambda }{\Delta \lambda }_{\mathrm{spec}}+CG_{A}R_{F}p_{0}P_{\mathrm{laser}}\frac{dn_{\mathrm{eff}}}{\mathrm{dp}}\frac{d\lambda }{dn_{\mathrm{eff}}}\frac{d^{2}T(\lambda )}{d\lambda^{2}}{\Delta \lambda }_{\mathrm{spec}}.$$

The shot noise originates from the discrete nature of photons and electrons. Its contribution to the final electronic noise is [43].

(8) $$V_{\mathrm{shot}}=\sqrt{2e(G_{A}T(\lambda_{E})P_{\mathrm{laser}}+G_{A}p_{0}SP_{\mathrm{laser}})\mathrm{MF}\left(x\right)∆f}R_{F},$$

where *M* is the gain of the PD, *e* is the charge of electrons, and $F\left(x\right)=M^{x}\;\left(0\leq x\leq 1\right)$ is the excess noise factor given the excess noise index *x*.

The Johnson noise generated by the thermal agitation of the charge carriers is defined as

(9) $$V_{\mathrm{Johnson}}=\sqrt{4k_{b}T∆fR_{F}}$$

where the $k_{b}$ is the Boltzmann constant, and the T is the temperature. Since all noise sources are uncorrelated, the total electric noise V_N_ is herein calculated as

(10) $$V_{N}=\sqrt{V_{\mathrm{RIN}}^{2}+V_{\mathrm{spec}}^{2}+V_{\mathrm{shot}}^{2}+V_{\mathrm{Johnson}}^{2}}$$

The final readout electric signal (V_total_) including all the noise terms is described as

(11) $$V_{\mathrm{total}}=V_{\mathrm{DC}}+V_{\mathrm{PA}}+V_{N}$$

Their contributions in a representative recorded voltage waveform are illustrated in Fig. 2a. We experimentally calibrated the correction coefficient *C* in Eq. (2) by using the experimental setup illustrated in Fig. 2b. The calibration setup consists of an acoustic source (V213-BC-RM, *Olympus*) being suspended 2 mm above the MRR ultrasound detector being attached to the bottom of a water tank. The acoustic source and the MRR are immersed in the water, which serves the purpose as the ultrasound coupling media in this study. The MRR was connected to the narrow-band laser and the matching electronics as shown in Fig. 1a. We first measured the acoustic pressure reference at the location of the MRR detector to be *p*_*0*_*=* 1142 Pa using a calibrated hydrophone (HNC-1000, *Onda*). We then replace it with the MRR for sensitivity calibration. The optical power of the narrow band laser (P_laser_) coupled into MRR is 2.83 µW. It was determined by measuring the optical transmission through the MRR at the off-resonance wavelengths. The recorded voltage output, the measured acoustic pressure *p*_*0*_, and other key process parameters are used in Eq. (2) and Eq . (5) to determine the coefficient *C*. The coefficient C is determined by the average ratio of the recorded PA voltage output from the measurement setup in Fig. 2b to the calculated PA signal without calibration, defined by $G_{A}R_{F}p_{0}P_{\mathrm{laser}}\mathrm{dT}(\lambda )/dp$, from Eq. (2) and Eq. (5). The coefficient *C* values are 912, 546, 622, and 390 for the Q-factor of 1 × 10^4^, 2 × 10^4^, 5 × 10^4^, and 1 × 10^5^, respectively. The calibration step allows our theoretical signal transduction model to closely represent the real experimental conditions.

## 3 Results

### 3.1 PA signal and noises from the comprehensive theoretical model

We use the calibrated signal transduction model to quantitatively investigate the influence of Q-factors and the driving wavelength on the voltage signals and the associated noises. The ratio of voltage signal and the noise will ultimately determine the nLOD for the MRR. Fig. 2c shows the calculated PA signal (V_PA_) for MRRs with varying Q-factors of 1 × 10^4^, 2 × 10^4^, 5 × 10^4^, and 1 × 10^5^. For the convenience of comparison, the wavelength in the horizontal axis shows the wavelength offset to its resonance being further normalized by the product of its resonance wavelength and the Q-factor (${Q\times \lambda }_{R}$). As such, the resonance curves of MRRs with different Q-factors are consolidated into a single Lorentzian form shown as the dash line in Fig. 2c. Since the sensitivity to the acoustic pressure is proportional to the slope of the resonance curve, $\mathrm{dT}(\lambda )/d\lambda_{R}$, as described in Eq. (2), the calculated V_PA_ has the maximum value at the normalized wavelength of ± 0.29/Q where the dT(λ)/dλ is maximized. The maximum value of V_PA_ is proportional to the Q-factor, as expected. Fig. 2d shows the calculation of all the resulting noise terms using Eq. (10). Specifically, V_RIN_ was calculated in Eq. (6) where RIN = 0.02 was determined using the S_RIN_ of the laser of − 120 dB and the ∆f of the 400 MHz from the system bandwidth limited by the bandwidth of PD [42]. V_spec_ was calculated in Eq. (7), where Δλ_spec_ of the laser source is 0.4 fm, the $\Delta f_{B}$ is 200 kHz [44], and the λ is 780 nm. V_shot_ was calculated using Eq. (8), where the *e* is 1.6 × 10^−19^ C, and the *M* is 100 and the *x* is 0.3 for avalanche PD at a wavelength of 780 nm [43]. V_Johnson_ was calculated using Eq. (9), where the $k_{b}$ is 1.38 × 10^−23^ J·K^−1^ and the T is the temperature 298.15 K for room temperature. The V_RIN_ from the laser power fluctuation and the shot noise V_shot_ are found to be the primary noise sources. In the case of an MRR with Q of 1 × 10^5^, V_RIN_ and V_shot_ have a peak value of 6.8 × 10^−2^ V and 4.2 × 10^−2^ V, respectively. In comparison, V_spec_ has a rather low peak value of 1.0 × 10^−4^ V due to the extremely low Δλ_spec_ of 0.4 fm in the narrow-band laser. V_Johnson_ has a constant value of 2.6 × 10^−4^ V and it is expected for commercial Si based avalanche PD modules with a ∆f of at least tens of MHz and an $R_{F}$ of at least a few kΩ. Similar behaviors are observed for all MRRs with different Q-factors (Fig. 2d). It is worthwhile to note that for an MRR with a higher Q-factor, V_N_ exhibits a distinct peak near the slope of the resonance spectra, which suggests a strong contribution from oscillating components of the voltage output (V_PA_). On the other hand, monotonic increase of V_N_ found in MRRs with lower Q-factor further suggests the strong contribution of the DC components of the voltage output (V_DC_).

### 3.2 Theoretical and experimental study for the detection limit

**Fig. 3:**
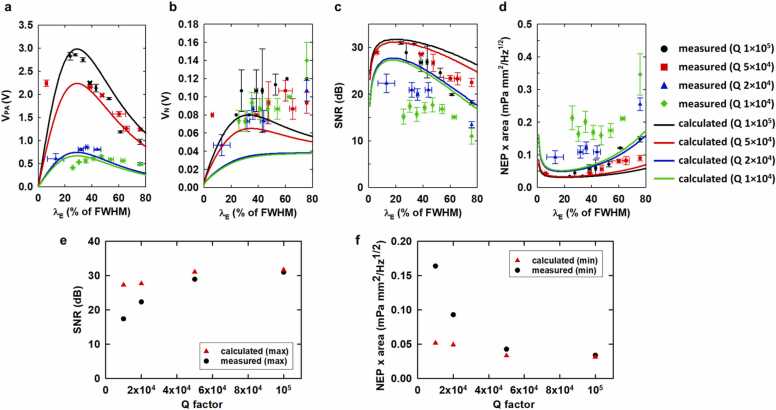
Theoretical and experimental results of V_PA_ (a), V_N_ (b), SNR (c), and nLOD (NEP × sensing area) (d) for MRRs with different Q-factors of 1 × 10^4^, 2 × 10^4^, 5 × 10^4^, and 1 × 10^5^. e) The optimal values of the theoretical and the experimental SNR for different Q-factors. f) The optimal values of the theoretical and the experimental nLOD for different Q-factors.

The detection limit of MRR based ultrasonic detectors ultimately relies on the competition among the signals and the noises, as both are proportional to the increase of the Q-factors. To better understand the detection limit, we experimentally measured V_PA_, V_N_, SNR, defined as V_PA_/V_N_, and the nLOD for MRRs with varying Q-factors and driving wavelength λ_E_ (Fig. 3). The NEP can be calculated by $V_{N}/(S\sqrt{∆f_{\mathrm{MRR}}})$, where $∆f_{\mathrm{MRR}}$ is the detection bandwidth of the MRR. The $∆f_{\mathrm{MRR}}$ and the sensing area of the MRR in the PACT system are 23 MHz [38] and 5 × 10^−3^ mm^2^ for the ring radius of 40 µm, respectively. The Q-factor of the MRR is fundamentally determined by the energy dissipation associated with the optical resonance. Specifically, it comprises the contribution of absorption loss, scattering loss, and the coupling loss. In this study, we intentionally perturbed the optimized device configuration and the nanofabrication conditions to create a set of MRRs with varying range of Q-factors from 1 × 10^4^ to 1 × 10^5^. Specifically, fabricated MRRs with a Q-factor of 1 × 10^4^ and 2 × 10^4^ using an UV-curable PDMS (KER-4690, *Shin-Etsu*) cladding/protection layer and MRRs with a Q-factor of 5 × 10^4^ and 1 × 10^5^ using a low refractive index (MY-131MC, *MY Polymer, Inc.*) cladding/protection layer. The range of Q-factors was chosen to cover the lower bound to the higher bound of Q-factors commonly seen in ultrasound detection. λ_E_ is expressed as the percentage of each FWHM, which can equivalently indicate the wavelength is how far away from the resonance dip of the MRR for each Q-factor. For example, 0% of FWHM represents the resonance dip.

Experimental results show good agreements with the theoretical model. The V_PA_ and V_N_ values tend to increase as the Q-factor increases because of the increase of the sensitivity (Fig. 3a and b). Both theoretical and experimental results tend to decrease as λ_E_ moves to the off-resonance region with lower $\mathrm{dT}(\lambda )/d\lambda_{R}$ resulting in lower sensitivity, while the calculated V_PA_ values have their maximum values at 29% of FWHM consistently in the theoretical model and the experimental V_PA_ values have the maximum at 47%, 36%, 6%, and 28% of FWHM for Q-factors of 1 × 10^4^, 2 × 10^4^, 5 × 10^4^, and 1 × 10^5^, respectively. In the theoretical model, the V_N_ values show two different trends depending on the Q-factor, one tends to gradually increase with the convergence value of 4.1 × 10^−2^ V, and the other tends to decrease with the same convergence value after having the maximum value, because the sensitivity term is converted to zero as λ_E_ moves away from the resonance curve. The experimental V_N_ values gradually increase as the λ_E_ increases similar to the theoretical model.

Both theoretical and experimental results clearly show that the higher Q-factor and an optimal λ_E_ at close to ∼20% of FWHM can provide higher SNR and lower nLOD in the PA measurement (Fig. 3c and d). The theoretical SNR has optimal values of 24.4, 24.9, 29.3, and 30.1 dB at the λ_E_ of 20%, 20%, 22%, and 23% of FHWM for Q-factors of 1 × 10^4^, 2 × 10^4^, 5 × 10^4^, and 1 × 10^5^, respectively. The experimental SNR has optimal values of 17.4, 22.3, 28.9, and 31 dB at λ_E_ of 30%, 13%, 6%, and 24% of FHWM for Q-factors of 1 × 10^4^, 2 × 10^4^, 5 × 10^4^, and 1 × 10^5^, respectively (Fig. 3e). The optimal nLOD for Q-factors of 1 × 10^4^, 2 × 10^4^, 5 × 10^4^, and 1 × 10^5^ are 7.21 × 10^−2^, 6.80 × 10^−2^, 4.13 × 10^−2^, and 3.75 × 10^−2^ mPa mm^2^/Hz^1/2^ in the theoretical results and 1.64 × 10^−1^, 9.30 × 10^−2^, 4.28 × 10^−2^, and 3.39 × 10^−2^ mPa mm^2^/Hz^1/2^ in the experimental results, respectively (Fig. 3f). Thus, the MRR can provide significantly improved nLOD, up to 177 times lower than nLOD of 6 mPa mm^2^/Hz^1/2^ from a commercialized piezoelectric transducer (V214-BB-RM, *Olympus NDT*) with a sensing area of 30 mm^2^ [16], thanks to the small size and the high detection sensitivity of MRR compared to piezoelectric transducers. The difference in absolute values and the optimal driving wavelengths between the theoretical and the experimental results is because the limitation of fine tuning of the wavelength of the narrow-band laser in the PA signal measurement unlike in the theoretical model.

### 3.3 PACT imaging with the MRR ultrasound detector

**Fig. 4:**
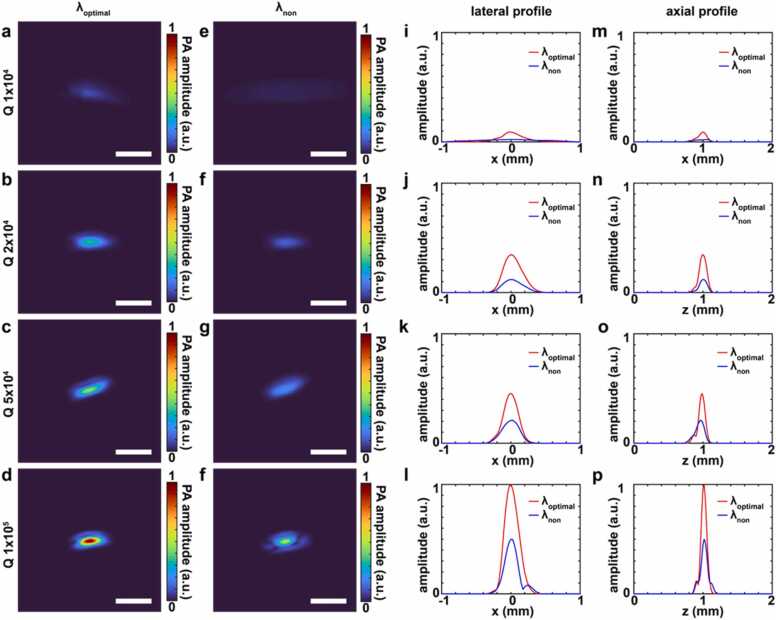
PACT imaging under the optimal and non-optimal driving wavelength conditions for MRRs with different Q-factors. **(a-d)** The normalized x-z slice images of the reconstructed 3D PACT images of a human hair sample under the optimal driving wavelength condition (λ_optimal_) for Q-factors of 1 × 10^4^, 2 × 10^4^, 5 × 10^4^, and 1 × 10^5^, respectively. Scale bar: 500 µm. **(e-f)** The normalized x-z slice images of the reconstructed 3D PA images of a human hair sample under non-optimal driving wavelength condition (λ_non_) for Q-factors of 1 × 10^4^, 2 × 10^4^, 5 × 10^4^, and 1 × 10^5^, respectively. Scale bar: 500 µm. **(i-l)** The normalized lateral signal profiles under λ_optimal_ and λ_non_ for Q-factors of 1 × 10^4^, 2 × 10^4^, 5 × 10^4^, and 1 × 10^5^, respectively. **(m-p)** The normalized axial signal profiles under λ_optimal_ and λ_non_ for Q-factors of 1 × 10^4^, 2 × 10^4^, 5 × 10^4^, and 1 × 10^5^, respectively.

We have also demonstrated PACT imaging of three human hair samples at varied depths using MRRs with different Q-factors at two different λ_E_ conditions to characterize PA imaging resolutions for different conditions of the MRR ultrasound detector (Fig. 4 and Fig. S1 in the Supporting Information). Two different λ_E_ close to the dip and far from the dip are chosen as the optimal (λ_optimal_) and the non-optimal driving wavelengths (λ_non_) to compare the highest and the lowest SNR conditions in PA imaging. In details, the λ_optimal_ is 30%, 13%, 6%, and 24% of FWHM for the Q-factor of 1 × 10^4^, 2 × 10^4^, 5 × 10^4^, and 1 × 10^5^, respectively, based on the experimental results of SNR in Fig. 3c. The λ_non_ is 76% of FWHM for all Q factors. The PACT images were acquired by using the MRR based PACT system described in Fig. 1a and reconstructed by using a 3D delay-and-sum algorithm [45], [46]. The optical energy was 10 mJ/pulse at 532 nm to image a human hair and a total imaging time was ∼1.6 s for a scanning distance of 1 mm with the laser repetition rate of 10 Hz and a scanning step size of 60 µm.

We acquired normalized x-z slice images of the reconstructed PACT images of the three human hair samples at varied depths for Q-factors of 1 × 10^4^, 2 × 10^4^, 5 × 10^4^, and 1 × 10^5^ and λ_E_ at λ_optimal_ and λ_non_ (Fig. S1 in the Supporting information). Each hair is located at z of ∼ 9 mm, ∼12.5 mm, and ∼15.4 mm. Enlarged reconstructed images of the hair sample at z of ∼15.4 mm, yellow dashed rectangular boxes in Fig. S1, for Q-factors of 1 × 10^4^, 2 × 10^4^, 5 × 10^4^, and 1 × 10^5^ are shown in Fig. 4a-d for λ_optimal_ and Fig. 4e-h for λ_non_. For the convenience of comparison, the x-z slice images are normalized by the maximum PA amplitude of the x-z image with a Q-factor of 1 × 10^5^ and λ_optimal_. The MRR with a Q-factor of 1 × 10^5^ provides clear images with a maximum normalized PA amplitude of 1 at λ_optimal_ (Fig. 4d), while the MRR with a Q-factor of 1 × 10^4^ shows blurred image with a maximum normalized PA amplitude of 0.09 (Fig. 4a). In contrast, the MRRs with the Q-factor of 1 × 10^4^ and 1 × 10^5^ provide a maximum normalized PA amplitude of 0.02 and 0.50 at λ_non_ (Fig. 4e and h), which are much lower than the maximum values from the optimal condition. The one-dimensional (1D) signal profiles in the lateral and axial directions are extracted from the normalized x-z slice images in Fig. 4a-h to compare the PA signal and signal contrast between the λ_optimal_ and λ_non_ conditions for different Q-factors. The lateral signal profiles in the x-direction (Fig. 4i-l) and the axial signal profiles in the z-direction (Fig. 4m-p) clearly indicate that the higher Q-factor and the λ_optimal_ condition can provide higher PA amplitude and signal contrast. In addition, we measured sizes of the human hair sample, defined by the FWHM of the lateral and the axial signal profiles in Fig. 4i-p, for different Q-factors and λ_E_ (Table 1). The measured lateral and axial sizes of the human hair sample are 308 µm and 136 µm, respectively, for a Q-factor of 1 × 10^4^ under the λ_optimal_ condition, i.e, λ_E_ at 30% of FWHM. Meanwhile, the measured sizes in the lateral and axial axes are 243 µm and 93 µm, respectively, for the Q-factor of 1 × 10^5^ under the λ_optimal_ condition, i.e, λ_E_ at 24% of FWHM. The spatial resolution of PACT is eventually determined by the SNR in the detected ultrasound signal, particularly in the high frequency band. The slope of the resonance dip is proportional to the Q-factor. as shown in Fig. 1d. It determines the magnification ratio when transducing the incident ultrasonic pressure wave into the temporally modulated electrical signal. Thus, a higher Q-factor is expected to produce a better detection sensitivity and thus a higher SNR in the detected ultrasound signal. The increased SNR will positively influence both the axial resolution ($R_{a}$) and the lateral resolution ($R_{l}$) in PACT, which are defined as: [7], [8], [9].

(12) $$R_{a}=0.6\lambda_{c}=0.6v_{m}/f_{c}$$

(13) $$R_{l}=\sqrt{R_{a}^{2}+D^{2}}$$

where $\lambda_{c}$ is the acoustic wavelength at the high cut-off frequency, $v_{m}$ is the speed of sound in the medium, $f_{c}$ is the cut-off frequency, and D is the diameter of the MRR. Higher SNR will improve the detection frequency bandwidth from the white noise background and thus, improve both the axial resolution and the lateral resolution.

**Table 1:** Measured lateral and axial sizes of the human hair sample.

Q-factor	lateral size (μm)		axial size (μm)	
	at λ_optimal_	at λ_non_	at λ_optimal_	at λ_non_
1 × 10^4^	308	1193	136	263
2 × 10^4^	320	347	126	130
5 × 10^4^	250	293	104	145
1 × 10^5^	243	210	93	96

## 4 Discussion

While the trends of V_PA_, V_N_, SNR, and nLOD along $\lambda_{E}$ exhibit consistent alignment with values of the same order in both the theoretical and experimental results for all Q-factors, some of the noise values show a difference in between the theoretical and experimental results. The errors in precisely modeling the contribution of different noise sources may collectively contribute to the apparent differences between the theoretical model and the experiment. One of the reasons can be the contribution of the excess noise factor $F\left(x\right)$ in the Eq. (8). We set an excess noise index x of 0.3 for $F\left(x\right)$ of V_shot_ in the Eq. (8) with assuming that the PD follows a $F\left(x\right)$-*M* relationship of a near infrared type [41], [43]. If an actual value of $F\left(x\right)$ of the PD is higher than 0.3, the V_shot_ value will increase thus, the V_N_ value increase. We compared theoretical results for two different excess noise indexes, 0.3 and 0.5, for the same conditions of other parameters described in the section above. V_shot_ values increase when the excess noise index x increases from 0.3 to 0.5. (Fig. S2 in the Supporting Information). When x is 0.3 as in the literature, the V_shot_ is lower than the V_RIN_ value for a Q-factor of 1 × 10^5^ and 5 × 10^4^ and the V_N_ and the V_RIN_ are almost same for a Q-factor of 2 × 10^4^ and 1 × 10^4^. When x is 0.5, the V_shot_ is similar to V_RIN_ for a Q-factor of 1 × 10^5^ and the V_N_ is higher than the V_RIN_ for a Q-factor of 5 × 10^4^, 2 × 10^4^ and 1 × 10^4^. As a result, V_N_, SNR, and nLOD values in the theoretical results are closer to the experimental results because the V_PA_ values are the same for different x values (Fig. S2 and S3 in the Supporting Information).

The minimum transmittance value at $\lambda_{R}$ can affect the output signal and noise, while the Q-factor of the MRR is the critical parameter to determine the performance of the ultrasound detector including the detection sensitivity, SNR, and nLOD based on the theoretical and experimental results. For the same $\lambda_{E}$, $\lambda_{R}$, and Q-factor, as the minimum transmittance at the resonance wavelength $T(\lambda_{R})$ increases, the slope of the resonance curve, dT(λ)/dλ, decreases and the transmittance at $T\left(\lambda_{E}\right)$ increases. The decrease of dT(λ)/dλ affects mainly V_PA_ and subsequently the V_N_. The decrease of dT(λ)/dλ induces the decrease of the detection sensitivity resulting in the decrease of V_PA_ for the same calibration parameter *C* based on (1), (2), (3), (4), (5). This further affects V_RIN_, V_spec_, and V_shot_ based on (6), (7), (8). While both $T\left(\lambda_{E}\right)$ and dT(λ)/dλ are related to the noise values, the change in dT(λ)/dλ is dominant. In the theoretical model, we assume that the normalized transmittance curve of the resonance spectrum of the MRR follows the Lorentzian form and $T(\lambda_{R})$ is 0.01, which is ideally close to zero for the critical coupling condition. As $T\left(\lambda_{R}\right)$ increases from 0.01 to 0.50, both V_PA_ and V_N_ values decrease for all Q-factors (Fig. S4 in the Supporting Information). The maximum value of V_PA_ for a Q-factor of 1 × 10^5^ is reduced by half and the maximum V_N_ decreases to 75% of the original value when $T\left(\lambda_{R}\right)$ increases from 0.01 to 0.50. Consequently, the SNR values decrease and the nLOD values increase for all Q-factors, which means performance of the MRR ultrasound detector is worsen. Therefore, the MRR ultrasound detector can provide the best detection sensitivity, SNR, and nLOD for higher Q-factor and the critical coupling condition under the optimal driving wavelength.

## 5 Conclusions

In conclusion, we developed a comprehensive theoretical model to comprehend the PA signal transduction process considering all the major noise factors in the MRR based ultrasound detectors. We quantitively studied the contribution of the major noise factors to the SNR and the ultrasound detection limit using both a theoretical model and experimental measurements. The Q-factor and the driving wavelength of the MRR directly related to the detection sensitivity are major parameters for the SNR and the nLOD in the system. The theoretical model and the experimental result clearly show that the higher Q-factor and the optimal driving wavelength exhibit optimal SNR and nLOD. Given an MRR with a Q-factor of 1 × 10^5^, the theoretical model predicts an optimal SNR of 30.1 dB and a corresponding nLOD of 3.75 × 10^-2^ mPa mm^2^/Hz^1/2^, which are in good agreement with the experimental measurements of 31.0 dB and 3.39 × 10^-2^ mPa mm^2^/Hz^1/2^, respectively. In addition, we demonstrated that a higher Q-factor MRR with the optimal driving wavelength can provide higher imaging resolution and contrast in PACT imaging. This work can provide guidance in understanding comprehensive PA signal transduction processes and contributions of major noises to optimize the SNR and nLOD in the MRR based PACT system, in attaining higher imaging resolution and contrast.

## Declaration of Competing Interest

C. S. and H. F. Z. have financial interests in Opticent Inc., which did not support this work. The other authors declare no conflict of interest.

## Data Availability

Data will be made available on request.

## References

[1] Wang L.V. (2009). Multiscale photoacoustic microscopy and computed tomography. Nat. Photonics.

[2] Wang L.H.V., Hu S. (2012). Photoacoustic tomography: in vivo imaging from organelles to organs. Science.

[3] Taruttis A., Ntziachristos V. (2015). Advances in real-time multispectral optoacoustic imaging and its applications. Nat. Photonics.

[4] Song K.H., Stoica G., Wang L.H.V. (2006). In vivo three-dimensional photoacoustic tomography of a whole mouse head. Opt. Lett..

[5] Maslov K., Zhang H.F., Hu S., Wang L.V. (2008). Optical-resolution photoacoustic microscopy for in vivo imaging of single capillaries. Opt. Lett..

[6] Wang L.H.V., Gao L. (2014). Photoacoustic microscopy and computed tomography: from bench to bedside. Annu Rev. Biomed. Eng..

[7] Xu M.H., Wang L.H.V. (2009). Analysis of spatial resolution in photoacoustic tomography. Opt. Sci. Eng. -Crc.

[8] Haltmeier M., Zangerl G. (2010). Spatial resolution in photoacoustic tomography: effects of detector size and detector bandwidth. Inverse Probl..

[9] Xia J., Yao J.J., Wang L.V. (2014). Photoacoustic Tomography. Princ. Adv., Prog. Electro Res.

[10] Park J., Park B., Kim T.Y., Jung S., Choi W.J., Ahn J., Yoon D.H., Kim J., Jeon S., Lee D., Yong U., Jang J., Kim W.J., Kim H.K., Jeong U., Kim H.H., Kim C. (2021). Quadruple ultrasound, photoacoustic, optical coherence, and fluorescence fusion imaging with a transparent ultrasound transducer. P Natl. Acad. Sci. USA.

[11] Park B., Han M., Park J., Kim T., Ryu H., Seo Y., Kim W.J., Kim H.H., Kim C. (2021). A photoacoustic finder fully integrated with a solid-state dye laser and transparent ultrasound transducer. Photoacoustics.

[12] Dangi A., Agrawal S., Kothapalli S.R. (2019). Lithium niobate-based transparent ultrasound transducers for photoacoustic imaging. Opt. Lett..

[13] Chen H.Y., Agrawal S., Dangi A., Wible C., Osman M., Abune L., Jia H.Z., Rossi R., Wang Y., Kothapalli S.R. (2019). Optical-resolution photoacoustic microscopy using transparent ultrasound transducer. Sens. -Basel.

[14] Ren D.Y., Sun Y.Z., Shi J.H., Chen R.M. (2021). A review of transparent sensors for photoacoustic imaging applications. Photonics.

[15] Dong B.Q., Sun C., Zhang H.F. (2017). Optical detection of ultrasound in photoacoustic imaging. IEEE T Bio-Med Eng..

[16] Wissmeyer G., Pleitez M.A., Rosenthal A., Ntziachristos V. (2018). Looking at sound: optoacoustics with all-optical ultrasound detection. Light-Sci. Appl..

[17] Zhang E., Laufer J., Beard P. (2008). Backward-mode multiwavelength photoacoustic scanner using a planar Fabry-Perot polymer film ultrasound sensor for high-resolution three-dimensional imaging of biological tissues. Appl. Opt..

[18] Plumb A.A., Huynh N.T., Guggenheim J., Zhang E., Beard P. (2018). Rapid volumetric photoacoustic tomographic imaging with a Fabry-Perot ultrasound sensor depicts peripheral arteries and microvascular vasomotor responses to thermal stimuli. Eur. Radio..

[19] Jathoul A.P., Laufer J., Ogunlade O., Treeby B., Cox B., Zhang E., Johnson P., Pizzey A.R., Philip B., Marafioti T., Lythgoe M.F., Pedley R.B., Pule M.A., Beard P. (2015). Deep in vivo photoacoustic imaging of mammalian tissues using a tyrosinase-based genetic reporter. Nat. Photonics.

[20] Rousseau G., Gauthier B., Blouin A., Monchalin J.P. (2012). Non-contact biomedical photoacoustic and ultrasound imaging. J. Biomed. Opt..

[21] Chow C.M., Zhou Y., Guo Y.B., Norris T.B., Wang X.D., Deng C.X., Ye J.Y. (2011). Broadband optical ultrasound sensor with a unique open-cavity structure. J. Biomed. Opt..

[22] Yakovlev V.V., Dickson W., Murphy A., McPhillips J., Pollard R.J., Podolskiy V.A., Zayats A.V. (2013). Ultrasensitive non-resonant detection of ultrasound with plasmonic metamaterials. Adv. Mater..

[23] Zhu X.Y., Huang Z.Y., Wang G.H., Li W.Z., Zou D., Li C.H. (2017). Ultrasonic detection based on polarization-dependent optical reflection. Opt. Lett..

[24] Liang Y.Z., Jin L., Wang L.D., Bai X., Cheng L.H., Guan B.O. (2017). Fiber-laser-based ultrasound sensor for photoacoustic imaging. Sci. Rep. -Uk.

[25] Liang Y.Z., Li L.X., Li Q., Liang H., Jin L., Wang L.D., Guan B.O. (2020). Photoacoustic computed tomography by using a multi-angle scanning fiber-laser ultrasound sensor. Opt. Express.

[26] Rosenthal A., Razansky D., Ntziachristos V. (2011). High-sensitivity compact ultrasonic detector based on a pi-phase-shifted fiber Bragg grating. Opt. Lett..

[27] Guggenheim J.A., Li J., Allen T.J., Colchester R.J., Noimark S., Ogunlade O., Parkin I.P., Papakonstantinou I., Desjardins A.E., Zhang E.Z., Beard P.C. (2017). Ultrasensitive plano-concave optical microresonators for ultrasound sensing. Nat. Photonics.

[28] Zhang E.Z., Beard P.C. (2011). A miniature all-optical photoacoustic imaging probe, Photons Plus. Ultrasound.: Imaging Sens..

[29] Shnaiderman R., Mustafa Q., Ulgen O., Wissmeyer G., Estrada H., Razansky D., Chmyrov A., Ntziachristos V. (2021). Silicon-photonics point sensor for high-resolution optoacoustic imaging. Adv. Opt. Mater..

[30] Shnaiderman R., Wissmeyer G., Ulgen O., Mustafa Q., Chmyrov A., Ntziachristos V. (2020). A submicrometre silicon-on-insulator resonator for ultrasound detection. Nature.

[31] Ling T., Chen S.L., Guo L.J. (2011). High-sensitivity and wide-directivity ultrasound detection using high Q polymer microring resonators. Appl. Phys. Lett..

[32] Ling T., Chen S.L., Guo L.J. (2011). Fabrication and characterization of high Q polymer micro-ring resonator and its application as a sensitive ultrasonic detector. Opt. Express.

[33] Li H., Dong B.Q., Zhang Z., Zhang H.F., Sun C. (2014). A transparent broadband ultrasonic detector based on an optical micro-ring resonator for photoacoustic microscopy. Sci. Rep. -Uk.

[34] Lee Y., Zhang H.F., Sun C. (2023). Highly sensitive ultrasound detection using nanofabricated polymer micro-ring resonators. Nano Converg..

[35] Dong B.Q., Li H., Zhang Z., Zhang K., Chen S.Y., Sun C., Zhang H.F. (2015). Isometric multimodal photoacoustic microscopy based on optically transparent micro-ring ultrasonic detection. Optica.

[36] Dong B.Q., Chen S.Y., Zhang Z., Sun C., Zhang H.F. (2014). Photoacoustic probe using a microring resonator ultrasonic sensor for endoscopic applications. Opt. Lett..

[37] Li H., Dong B.Q., Zhang X., Shu X., Chen X.F., Hai R.H., Czaplewski D.A., Zhang H.F., Sun C. (2019). Disposable ultrasound-sensing chronic cranial window by soft nanoimprinting lithography. Nat. Commun..

[38] Rong Q., Lee Y., Tang Y., Vu T., Taboada C., Zheng W., Xia J., Czaplewski D.A., Zhang H.F., Sun C., Yao J. (2022). High-frequency 3D photoacoustic computed tomography using an optical microring resonator. BME Front..

[39] Chao C.Y., Ashkenazi S., Huang S.W., O'Donnell M., Guo L.J. (2007). High-frequency ultrasound sensors using polymer microring resonators. Ieee T Ultrason Ferr..

[40] Bogaerts W., De Heyn P., Van Vaerenbergh T., De Vos K., Selvaraja S.K., Claes T., Dumon P., Bienstman P., Van Thourhout D., Baets R. (2012). Silicon microring resonators. Laser Photonics Rev..

[41] Thorlabs, APD430x Mannual, 2020. https://www.thorlabs.com/drawings/c801244f0fb67e63–9DEFECC1-B0CF-3^82^B–^67^C4DC84C4690BA2/APD430A2_M-Manual.pdf.

[42] Zhou X.Y., Zhang L., Pang W. (2016). Performance and noise analysis of optical microresonator-based biochemical sensors using intensity detection. Opt. Express.

[43] H.P.K.K. , Si APD Technical Note, 〈https://www.hamamatsu.com/content/dam/hamamatsu-photonics/sites/documents/99_SALES_LIBRARY/ssd/si-apd_kapd9007e.pdf〉 (2021).

[44] Newport, Velocity TLB-6700, 2017. 〈www.newport.com/medias/sys_master/images/images/hcd/hd6/9123119824926/SP-NF-DS-20171024-Velocity6700.pdf〉.

[45] Mozaffarzadeh M., Mahloojifar A., Orooji M., Adabi S., Nasiriavanaki M. (2018). Double-stage delay multiply and sum beamforming algorithm: application to linear-array photoacoustic imaging. Ieee T Bio-Med Eng..

[46] Kang H., Lee S.W., Lee E.S., Kim S.H., Lee T.G. (2015). Real-time GPU-accelerated processing and volumetric display for wide-field laser-scanning optical-resolution photoacoustic microscopy. Biomed. Opt. Express.

