# Supplementary material for: Theoretical and experimental study on the detection limit of the micro-ring resonator based ultrasound point detectors
Source: Photoacoustics. 2023 Nov 29;34:100574. doi: 10.1016/j.pacs.2023.100574 (PMC10731384; doi:10.1016/j.pacs.2023.100574)
Supplement: Supplementary file 1 — Supplementary material [file mmc1.pdf]

Supporting Information

Theoretical and Experimental Study on the Detection Limit of the Micro-ring Resonator Based Ultrasound Point Detectors

Youngseop Lee,[1,2,ǂ] Qiangzhou Rong,[3,ǂ] Ki-Hee Song,[1] David A. Czaplewski,[4] Hao F Zhang,[1] Junjie Yao,[3,*] Cheng Sun[2,**]

[1]*Department of Biomedical Engineering, Northwestern University, Evanston, IL 60208, USA*
[2]*Department of Mechanical Engineering, Northwestern University, Evanston IL 60208, USA*
[3]*Department of Biomedical Engineering, Duke University, Durham, NC 27708, USA*
[4]*Center for Nanoscale Materials, Argonne National Laboratory, Argonne, IL 60439, USA*
**junjie.yao@duke.edu*
***c-sun@northwestern.edu*

ǂThese authors equally contributed to this work.

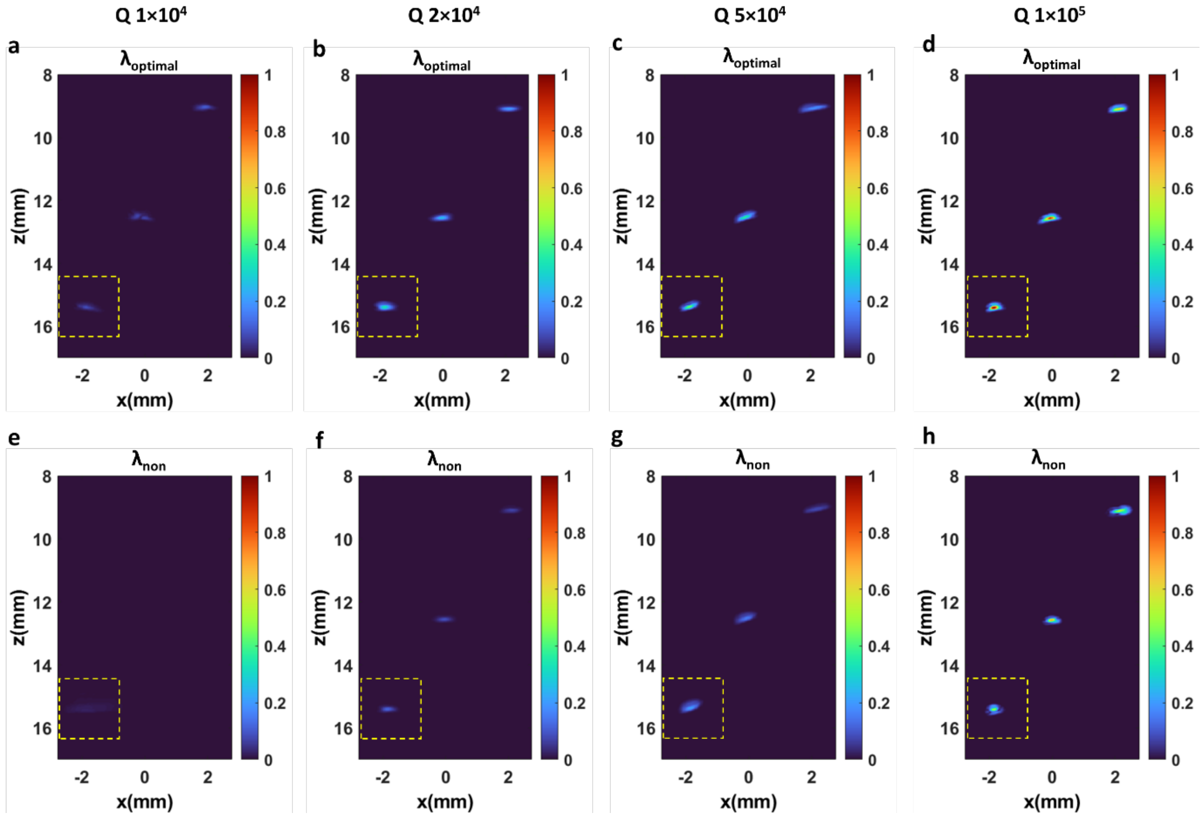

Fig. S1. Normalized PACT images of three human hairs at varied depths under the optimal and non-optimal driving wavelength conditions for MRRs with different Q-factors. **(a-d)** The normalized x-z slice images of the reconstructed 3D PACT images of the human hair sample under the optimal driving wavelength condition ($\lambda_{optimal}$) for Q-factors of 1×10^4, 2×10^4, 5×10^4, and 1×10^5, respectively. **(e-f)** The normalized x-z slice images of the reconstructed 3D PA images of the human hair sample under non-optimal driving wavelength condition (λ_{non}) for Q-factors of 1×10^4, 2×10^4, 5×10^4, and 1×10^5, respectively. Note that the enlarged images and the 1D lateral/axial profiles of the yellow dashed rectangular boxes are displayed in the manuscript.

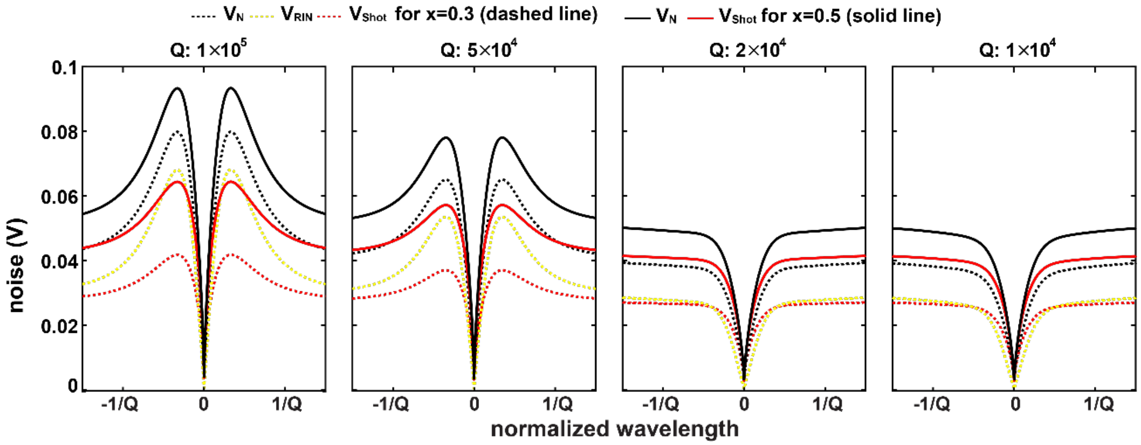

Fig. S2. The comparison of theoretically calculated V_{shot} and V_N values for two different excess noise indexes, 0.3 and 0.5, for all Q-factors. Dashed lines are for the theoretical results for the excess noise index of 0.3 and solid lines are for the theoretical results for the excess noise index of 0.5. Note that the V_{RIN} (a yellow dash line) is displayed for comparison.

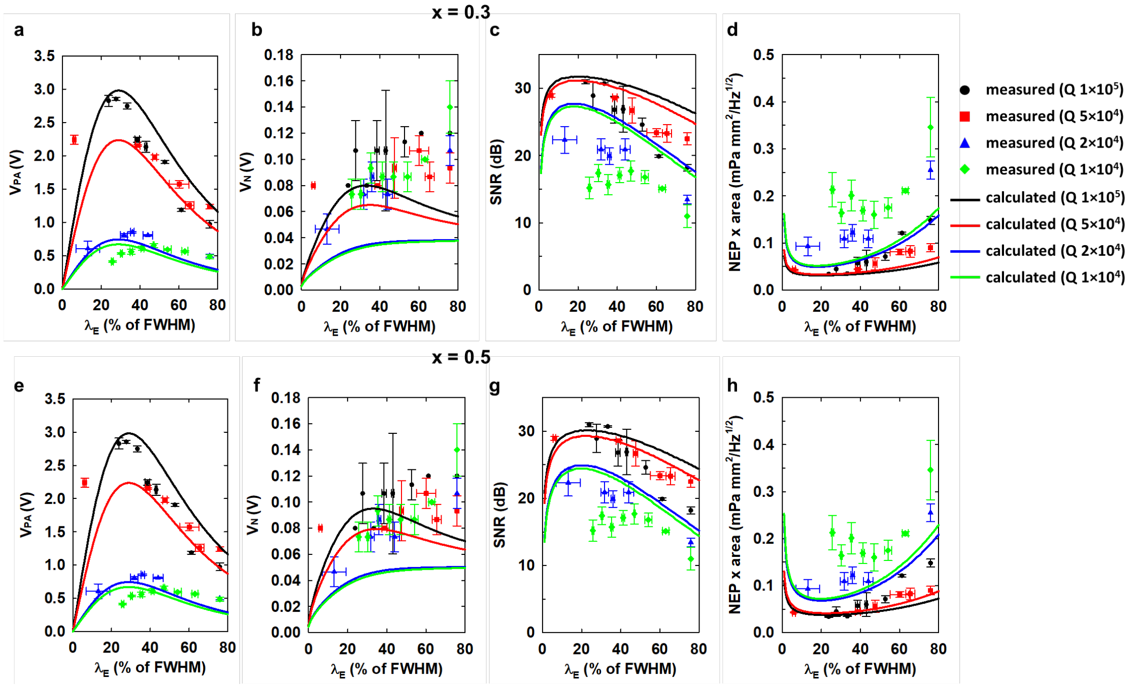

Fig. S3. Comparison of theoretical results for different excess noise indexes. **(a-d)** Theoretical and experimental results of VPA, VN, SNR, and nLOD (NEP × area) for an excess noise index of 0.3. **(e-h)** Theoretical and experimental results of VPA, VN, SNR, and nLOD (NEP × area) for an excess noise index of 0.5.

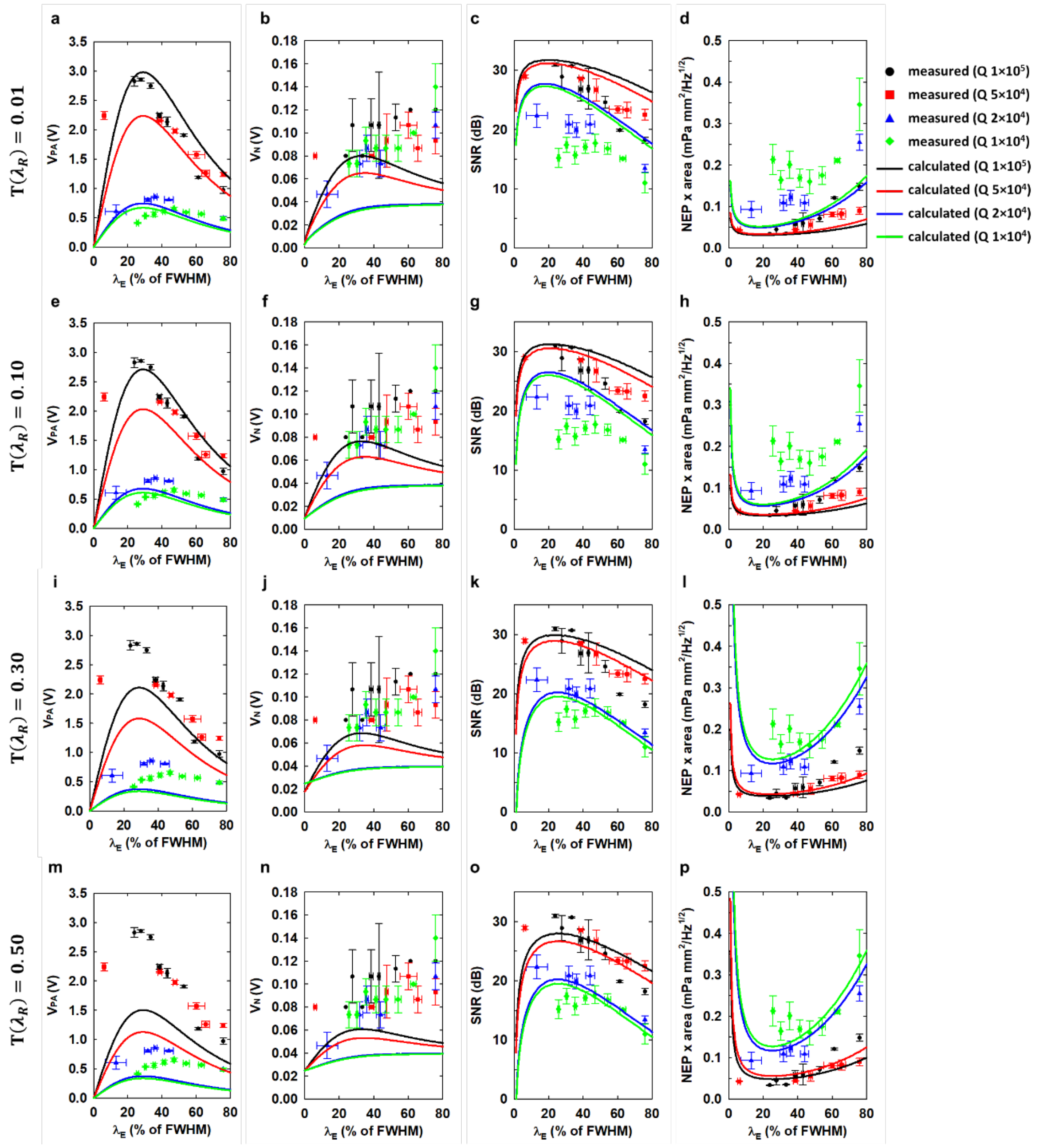

Fig. S4. Comparison between the theoretical and experimental results for different $T(\lambda_R)$ values of 0.01 (**a-d**, the original graphs in the manuscript), 0.10 (**e-h**), 0.30 (**i-l**), and 0.50 (**m-p**).
